# Multimorbidity prevalence and patterns across socioeconomic determinants: a cross-sectional survey

**DOI:** 10.1186/1471-2458-12-201

**Published:** 2012-03-19

**Authors:** Calypse B Agborsangaya, Darren Lau, Markus Lahtinen, Tim Cooke, Jeffrey A Johnson

**Affiliations:** 1 Department of Public Health Sciences, 2-040 Li Ka Shing Center for Health Research and Innovation, University of Alberta, Edmonton, Alberta T6G 2E1, Canada; 2 Health Quality Council of Alberta, Calgary, Alberta, Canada

## Abstract

**Background:**

Studies on the prevalence of multimorbidity, defined as having two or more chronic conditions, have predominantly focused on the elderly. We estimated the prevalence and specific patterns of multimorbidity across different adult age groups. Furthermore, we examined the associations of multimorbidity with socio-demographic factors.

**Methods:**

Using data from the Health Quality Council of Alberta (HQCA) 2010 Patient Experience Survey, the prevalence of self reported multimorbidity was assessed by telephone interview among a sample of 5010 adults (18 years and over) from the general population. Logistic regression analyses were performed to determine the association between a range of socio-demographic factors and multimorbidity.

**Results:**

The overall age- and sex-standardized prevalence of multimorbidity was 19.0% in the surveyed general population. Of those with multimorbidity, 70.2% were aged less than 65 years. The most common pairing of chronic conditions was chronic pain and arthritis. Age, sex, income and family structure were independently associated with multimorbidity.

**Conclusions:**

Multimorbidity is a common occurrence in the general adult population, and is not limited to the elderly. Future prevention programs and practice guidelines should take into account the common patterns of multimorbidity.

## Background

Multimorbidity, the concurrent occurrence of two or more chronic conditions [1], is increasingly common, probably due to aging populations, lowered threshold of diagnosis, inclusion of traditional risk factors such as obesity into its definition, longevity achieved through advances in medical care or possibly a true increase in the prevalence of some chronic diseases [2].

As in other industrialized countries, Canadian healthcare delivery is typically guided by clinical practice guidelines that are oriented towards single-diseases [3]. This poses a challenge for primary care professionals who try to implement evidence from these guidelines in caring for patients with multimorbidity. Individuals with multimorbidity are therefore at increased risk of receiving less than best practice care [4,5], more frequent and longer hospitalizations, higher health care costs and increased use of polypharmacy with the potential for adverse drug effects [6]. The challenges have prompted calls for patient care guidelines and health programs that are multiple disease-centered [2,7,8].

Furthermore, from a public health perspective, surveillance systems for chronic diseases tend to focus on single conditions. In Canada, for example, the National Diabetes Surveillance System (NDSS) was developed to track diabetes incidence, prevalence and mortality in all provinces and territories [9]. In the province of Alberta, this system has been embellished to report more extensively on a variety of comorbidities in people with diabetes [10], but it remains focused on a single, albeit common, condition in the population. The Public Health Agency of Canada has recently expanded the model from the NDSS to provide surveillance data on other conditions under the umbrella of the Canadian Chronic Disease Surveillance System (CCDSS), but this approach still retains the single disease focused model, with some attention to relevant comorbidities. Given that several common chronic conditions may cluster as multimorbidity in the general population, it would seem appropriate to take a multimorbidity approach to population health surveillance. Moreover, given a common set of shared risk factors (e.g., smoking, obesity, physical activity), multimorbidity surveillance may be more appropriate to evaluate the efficiency of more general or broader public health interventions.

Estimates of the prevalence of multimorbidity vary from 17% to over 90% [1,11,12]. The wide variation is due to dissimilar study populations or data sources, usually entailing differences in demographic characteristics and disease types or classification [11-13]. Most studies have been limited to patients in the primary care setting [1,14-17], having a specific index disease [18-21] or to just the elderly [13,22-25]. Few studies have evaluated the prevalence of multimorbidity across age groups of the general population, including younger adults [11,26].

In a recent study on the prevalence of multimorbidity in a population-based cohort in South Australia [11], the authors concluded that multimorbidity is not just a condition of the elderly. However, the definition of multimorbidity in their study was based on participants having two or more of a limited number of chronic conditions; asthma, cardiovascular disease, chronic obstructive pulmonary disease, diabetes, a mental health condition, arthritis and osteoporosis. Validity of the prevalence estimates potentially increases when a broader list of common chronic conditions is included in the study.

In Canada, for example, a study by Fortin and colleagues [26] observed an overall prevalence of 11.6% in the general population and 32.5% in practice-based population, using data obtained from adults (25+ years) in the province of Quebec. This study highlighted the higher prevalence of multimorbidity across different age groups in the primary care sample compared to the general population. However, Fortin and colleagues did not elaborate the particular clusters of chronic conditions that comprise the patterns of multimorbidity. Indeed, there are currently no published Canadian data on the specific patterns of multimorbidity combinations in the general population. Therefore, the aim of this study was to estimate the prevalence and patterns of multimorbidity in different adult age groups, as well as determine the association of multimorbidity with socio-demographic factors.

## Methods

The study is based on data from the Health Quality Council of Alberta (HQCA) 2010 Patient Experience Survey [27]. The survey evaluated a sample of adult Albertans, representative of the general adult population, on their experience and satisfaction with the quality of health services they receive. The survey instrument, a telephone-based questionnaire, was administered by Random-Digit Dialing (RDD) approach to ensure that households in each of the five health zones had an equal chance to be contacted. Data were collected from 5010 adult (≥ 18 years of age) respondents during the fall of 2010. Sampling weights were derived to account for the stratified sampling approach, with a provincially representative sample of the population across the five health zones of Alberta.

To determine the occurrence of chronic conditions, respondents were asked the question "*Do you have any of the following chronic conditions or diseases?"; *diabetes, chronic obstructive pulmonary disorder, asthma, hypertension, high cholesterol, sleep apnea, congestive heart failure, obesity, depression or anxiety, chronic pain, arthritis, heart disease, stroke (or related conditions) and cancer. Apart from these 14 chronic conditions, respondents were also asked to report any other chronic conditions not listed. Based on the most frequent responses to the open-ended "other conditions" query, two additional chronic conditions were identified: gastro-intestinal tract (GIT disease) and kidney diseases. This study, therefore, considered a total of 16 chronic conditions.

Disease status was based on self-reports, and multimorbidity was defined as the presence of two or more chronic conditions [1,15]. Demographic data were also based on self-report, and included respondents' age, sex, educational level, annual household income level, number of adults (≥ 18 years of age) and number of children (≤ 16 years of age) living in the same household.

We undertook primarily a descriptive statistics evaluation of multimorbidity in this analysis. The prevalence of multimorbidity was estimated in relation to age, sex, household income and educational level. Educational level was collapsed into three categories; high school (at most high school education), college (more than high school education, including completion of college education), and university (at least university degree). All prevalence measures were direct- standardized to the 2006 age and sex distribution of the Alberta population (2006 Canadian Census). We were also interested to study the most common clusters of chronic conditions. We therefore determined the most common pairs, triads, quartets and quintets of chronic conditions. These were assessed by clustering the disease types per individual, then reporting the most common (frequencies) within pairs, triads, etc. Data are reported only for combinations of chronic conditions that occurred six or more times in the sample [11].

Univariate logistic regression models were then used to evaluate the association between socio-demographic variables and multimorbidity within age strata (i.e. 18-24, 25-44, 45-64, 65 + years). Variables that were statistically significant (*p *= 0.05) within at least one age group were entered into a multivariate regression analysis, to examine the factors independently associated with multimorbidity. Analyses were adjusted for survey sampling weights. Statistical analyses and data management were performed using STATA V11 package. The Health Research Ethics Board (HREB) at the University of Alberta approved the data collection protocols and instruments.

## Results

### Study sample

The survey included 5010 respondents. Thirty (0.6%) respondents were excluded from the analysis due to missing chronic conditions data, leaving a sample of 4980 respondents in this study (Table 1). Representing the province's population, the sample had more females than males, average age of 46.7 (SD 16.5) years. The majority of respondents (52.8%) had gone on to secondary education. A substantial proportion of respondents 1677 (33.7%) reported at least one of the study chronic conditions, with an overall average of 2.3 (SD 1.7) (Table 1).

**Table 1:** Demographic characteristics of sample respondents

Variables	N (%)	Mean chronic conditions (SD)	Multimorbidity prevalence (%)	
			Crude	Adjusted (95% CI)
Sex				
Males	2393 (47.7)	2.3 (1.7)	16.8	15.6 (14.2-16.9)
Females	2618 (52.3)	2.3 (1.7)	20.6	19.2 (17.8-20.6)
^2^Age Group				
18-24	459 (9.2)	1.4 (0.9)	2.4	2.4 (1.0-3.9)
25-44	1934 (38.6)	1.7 (1.1)	9.3	9.3 (8.0-10.6)
45-64	1841 (36.8)	2.5 (1.7)	25.5	25.5 (23.5-27.5)
65+	776 (15.5)	2.8 (1.9)	36.2	35.8 (32.4-39.1)
^3^Health Zones				
South	892 (17.8)	2.5 (1.9)	19.6	17.4 (15.0-19.7)
Calgary	960 (37.0)	2.2 (1.5)	17.3	16.6 (14.4-18.9)
Central	893 (17.8)	2.5 (1.8)	19.9	17.3 (15.0-19.6)
Edmonton	978 (19.5)	2.2 (1.6)	19.8	18.7 (16.4-21.0)
North	1287 (25.7)	2.2 (1.7)	17.9	17.6 (15.6-19.6)
^3^Education				
High School	2367 (47.3)	2.5 (1.8)	20.8	18.5 (17.0-19.9)
College	1432 (28.6)	2.2 (1.5)	18.6	18.0 (16.1-19.9)
University	1211 (24.2)	2.1 (1.6)	15.2	14.5 (12.6-16.3)
^3^Income				
< $30,000	593 (13.7)	2.9 (2.1)	32.5	28.5 (24.6-32.4)
$30,000-59,000	1028 (23.7)	2.4 (1.6)	23.0	19.4 (17.2-21.6)
$60,000-99,000	1244 (28.7)	2.1 (1.6)	16.9	17.3 (15.1-19.5)
> = $100,000	1466 (33.9)	1.9 (1.3)	11.6	11.8 (9.5-14.0)

^1^Age-standardized ^2^Sex-standardized ^3^Age- and Sex-standardized (for adjusted prevalence)

### Prevalence of multimorbidity

The age and sex standardized prevalence of multimorbidity was 19.0% (95% CI 18.0-20.0) in the surveyed general adult population, and 49.1% (46.4-51.7) among those with at least one chronic condition. Of those with at least one chronic condition, there were 384 (22.9%), 214 (12.8%), 150 (8.9%) and 188 (11.2%), with two, three, four and five or more of the select chronic conditions, respectively. Thus, 7.7% of the population had 2 chronic conditions, 4.3% had 3, 3.0% had 4 and 3.8% had 5 or more concurrent chronic conditions.

**Figure 1:**
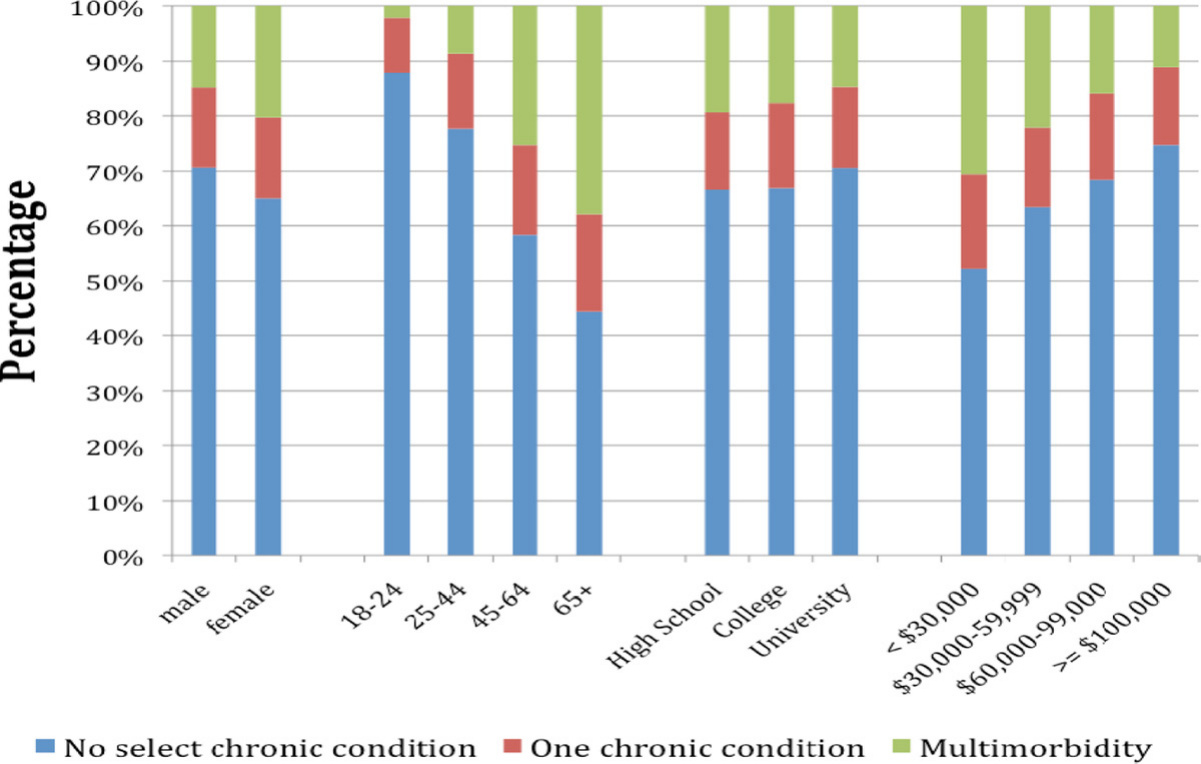
**The percentage adult population with (and without) multimorbidity by sex, age group, educational level, and household income of the Health Quality Council of Alberta 2011 survey^1^**.

The age-standardized prevalence of multimorbidity was higher in females (19.2%, 95% CI 17.8-20.6) than in males (15.6%, 95% CI 14.2-16.9). The prevalence increased steadily with age, being 2.4% in those younger than 25 years, 9.3% in those aged 25-44 years, a quarter of the population aged 45-64 years and one in every three of those aged 65 years or older (Table 1). Interestingly, 70.2% (657) of those with multimorbidity were less than 65 years of age (results not tabled). The prevalence of multimorbidity was lower in individuals reporting higher income (Table 1). Figure 1 illustrates the morbidity status of the population within each category of sex, age group, education and income.

### Common multimorbidity combinations

Among respondents with any two concurrent chronic conditions, the combination of arthritis and chronic pain was the most common (14.1%) (Table 2), and also among respondents aged less than 65 years of age, whereas hypertension and arthritis was the most common for those aged 65+ years. Among patients having three, four and five or more chronic conditions, the most common multimorbidity combinations were depression/anxiety- arthritis- chronic pain (9.4%), hypertension- depression/anxiety-arthritis- chronic pain (5.4%), and diabetes- hypertension- high cholesterol- arthritis-chronic pain, respectively (6.8%). The highest frequency in any multimorbidity disease combination was only n = 54, evidence of the marked variation in chronic disease combinations.

**Table 2:** Combination of chronic conditions among respondents with multimorbidity^1^

Disease cluster		
*Disease pairs (n = 384)*	**Frequency**	**Percent**
Chronic pain-Arthritis	54	14.1
Hypertension-Arthritis	29	7.6
Hypertension- High cholesterol	26	6.8
Depression/Anxiety -Chronic pain	24	6.3
Diabetes-Hypertension	20	5.2
Asthma-Depression/Anxiety	12	3.1
High cholesterol-Arthritis	12	3.1
Asthma-Chronic pain	11	2.9
Sleep apnea-Chronic pain	11	2.9
Hypertension-Chronic pain	10	2.3
Diabetes-Arthritis	9	2.3
Hypertension-Obesity	9	2.3
High cholesterol-Chronic pain	9	2.3
Hypertension-Depression/Anxiety	8	2.1
Hypertension-Cancer	8	2.1
Depression/Anxiety-Arthritis	8	2.1
*Disease triads (n = 214)*		
Depression/Anxiety- Chronic pain-Arthritis	20	9.4
Hypertension-Chronic pain-Arthritis	15	7.0
Hypertension-High cholesterol-Arthritis	13	6.1
Diabetes-Hypertension-High cholesterol	11	5.1
High cholesterol-Chronic pain-Arthritis	8	3.7
*Disease quartets (n = 149)*		
Hypertension-Depression/Anxiety-Chronic pain-Arthritis	8	5.4
Hypertension-High cholesterol-Chronic pain-Arthritis	6	4.0
*Disease quintets (n = 88)*		
Diabetes- Hypertension- High cholesterol-chronic pain- Arthritis	6	6.8
Asthma-Hypertension-obesity-Chronic pain-arthritis	6	5.9

^1 ^Only disease combinations with a frequency of at least 6 are listed

### Correlates of multimorbidity

Univariate logistic models showed that multimorbidity was significantly different across several socio-economic factors (Table 3). Age, sex, income, education and family structure were entered into the multivariate regression model. The female sex, older age, lower household income and NOT living with children under the age of 16 years, were independently associated with elevated odds of having multimorbidity (Table 4). After adjusting for the other factors, education was not a strong predictor of multimorbidity status.

**Table 3:** Univariate analysis of the association between demographic characteristics and multimorbidity by age groups^1^

	Age 18-24	Age 25-44	Age 45-64	Age 65+
**Variables**	**OR (95%CI), n = 455**	**OR (95%CI), n = 1921**	**OR (95%CI), n = 1833**	**OR (95%CI), n = 771**

Sex				
Males	1.00	1.00	1.00	
Females	2.22 (0.67-7.38)	1.40 (0.98-1.98)	1.49 (1.16-1.91)	1.78 (1.25-2.53)
Health Zones				
South	1.00	1.00	1.00	1.00
Calgary	1.16 (1.59-8.51)	0.61 (0.36-1.05)	1.05 (0.74-1.50)	1.12 (0.69-1.82)
Central	0.46 (0.04-5.16)	0.89 (0.51-1.56)	1.04 (0.71-1.51)	0.97 (0.60-1.56)
Edmonton	0.78 (0.12-5.64)	0.94 (0.57-1.56)	1.03 (0.73-1.50)	1.53 (0.97-2.41)
North	2.03 (0.36-11.94)	0.90 (0.56-1.45)	1.06 (0.76-1.49)	1.08 (0.68-1.73)
Education				
University	1.00	1.00	1.00	1.00
College	-	1.21 (0.75-1.97)	1.33 (0.94-1.88)	1.74 (0.99-3.06)
High school (or less)	1.47 (0.28-7.67)	1.31 (0.82-2.07)	1.45 (1.05-1.99)	1.24 (0.77-1.97)
Income				
> = $100,000	1.00	1.00	1.00	1.00
$60,000-99,999	0.85 (0.13-5.53)	1.79 (1.12-2.97)	1.47 (1.03-2.08)	2.49 (0.92-6.79)
$30,000-59,999	-	2.03 (1.15-3.57)	1.94 (1.35-2.78)	2.84 (1.11-7.27)
< $30,000	1.08 (0.15-7.96)	4.43 (2.34-8.37)	2.88 (1.85-4.48)	3.07 (1.19-7.86)
Family Structure				
Living with adults				
Yes	1.00	1.00	1.00	1.00
No	0.73 (0.14-3.90)	1.25 (0.77-2.05)	1.08 (0.80-1.45)	1.58 (0.79-3.19)
Living with children				
Yes	1.00	1.00	1.00	1.00
No	13.04 (1.59-106.76)	1.95 (1.34-2.83)	2.36 (1.59-3.51)	4.53 (1.28-16.02)

^1^Multimorbidity is defined as having two or more select chronic conditions. Point estimates are derived from a univariate regression (without adjusting for other factors). Age in years.

**Table 4:** Multivariate analysis of the association between socio-demographic characteristics and multimorbidity^1^

Variable	Odds ratio	95% Confidence Intervals	**P**^**trend**^
*Sex*			
Males	1.00	1.00	
Females	1.42	1.16-1.73	
*Age groups (years)*			
18-24	1.00		
25-44	7.59	3.29-17.51	
45-64	20.13	8.78-46.09	
65+	23.04	9.96-53.34	0.007
*Education*			
University	1.00		
College	1.07	0.82-1.40	
High school (or less)	1.11	0.86-1.42	
*Income*			
> = $100,000	1.00		
$60,000- 99,999	1.53	1.16-2.01	
$30,000- 59,999	1.78	1.35-2.36	
< $30,000	2.39	1.72-3.33	0.041
*Living with Children*			
Yes	1.00		
No	2.11	1.60-2.78	

^**1**^All variables are adjusted for each other in the multivariate regression. Multimorbidity is defined as the presence of two or more select chronic conditions.

Results of the multivariate analysis by age strata are illustrated in Table 5. Among persons aged 25-44 years, those more likely to have multimorbidity were: reporting an annual household income less than $100,000; and those NOT living with children aged 16 years or younger. Among those aged 45-64, multimorbidity was significantly more likely among females; those reporting a household income of less than $100,000; and those NOT living with children under 16 years of age. Finally, among persons aged 65 or more, multimorbidity was significantly more common among females; and those NOT living with children under 16 years of age.

**Table 5:** Multivariate analyses of the associations between socio-demographic factors and multimorbidity by age groups^1^

Variables	**Age 25 **- **44**	Age 45-64	Age 65+
	**OR (95% CI)**	**OR (95% CI)**	**OR (95% CI)**

*Sex*			
Males	1.00	1.00	1.00
Females	1.43 (0.92-2.20)	1.41 (1.06-1.86)	1.55 (1.03-2.33)
*Income*			
> = $100,000	1.00	1.00	1.00
$60,000-99,999	1.68 (1.01-2.80)	1.38 (1.00-1.97)	2.36 (0.85-6.56)
$30,000-59,999	1.84 (1.03-3.29)	1.69 (1.17-2.45)	2.50 (0.96-6.48)
< $30,000	3.72 (2.00-7.01)	2.44 (1.53-3.87)	2.62 (1.00-6.92)
*Living with Children*			
Yes	1.00	1.00	1.00
No	2.00 (1.29-3.02)	1.96 (1.30-2.95)	8.45 (2.02-35.41)

^1^Age group 18-24 has been excluded because of empty cells due to small sample sizes across strata

## Discussion

This study, based on a selection of community dwelling Albertans, describes the epidemiology of multimorbidity. The overall prevalence of multimorbidity in the study population was 19% in the general adult population. Age, household income and family structure were the most important measured predictors of the multimorbidity status. Multimorbidity tended to be more common in females than in males, an observation made in previous studies [23,26].

Our estimates of the overall prevalence of multimorbidity is comparable to studies in Quebec, Canada [26] and Australia [12], and lower than reports from hospital-based practice [12,28]. Patients consulting at a hospital for a chronic condition are more likely to have another chronic condition. In the Canadian study, Fortin and colleagues [26] compared the prevalence of multimorbidity in practice-based and general population samples in Quebec. They observed that the overall prevalence was significantly higher for the primary care sample (32.3%) than in the general population (11.6%), highlighting the importance of the study population characteristics in the interpretation of findings on the prevalence of multimorbidity. Their study, however, defined multimorbidity based on 7 chronic conditions in adults aged 25 years and over. The lower prevalence of multimorbidity for the general population observed in their study compared to the present study may be due to the limited number of chronic conditions included [26].

Studies examining the prevalence of multimorbidity have largely been limited to the elderly [13,22,25,29,30], indicating that multimorbidity is a condition of old age. We observed, however, that 70% of persons with multimorbidity were less than 65 years of age, consistent with previous observations that multimorbidity affects not just older people [11]. Mercer and colleagues [15] argued that future studies "must begin to investigate multimorbidity across a life-course". Our findings provide further evidence on the importance of multimorbidity in young adults.

There have recently been calls for a more holistic definition of the term, with the inclusion of not just chronic disease "labels" [15], but also morbidities suggesting emotional and psychological distress. The present study included anxiety and depression as a morbidity. The inclusion of another important chronic condition, obesity, remains controversial and has been considered elsewhere as a risk factor of multimorbidity, rather than a disease on its own right [11]. Nagel and colleagues [31] in a prospective study noted that obesity rates increase with the number of chronic conditions. While the direction of the relationship between obesity and multimorbidity is yet to be ascertained, there is need for public health policy to emphasize the importance of a healthy weight in reducing the burden of multimorbidity.

Age, household income and family structure (Not living with children) were independently association with multimorbidity. Although there is ample evidence for the inverse association between increasing age and decreasing income with multimorbidity [11,16], the importance of family structure has received little attention in the past. Taylor and colleagues [11] showed that independent of age, multimorbidity was more common among adults living alone or with partner, compared to those living with children. The reasons underlying these findings are not clearly understood. There is evidence that family support, also known as family-centered care [32], may be vital in the management and control of chronic diseases [33,34]. The importance of family support, through chronic disease management, may be an important component in reducing the likelihood of developing other chronic conditions. However, this hypothesis remains to be tested.

A major strength of this study is the population representativeness of the study sample, that allows for generalization of the findings. Thus, findings represent prevalence estimates in the general adult population. Population-based prevalence estimates of multimorbidity are important for reporting about the health status of the population. Our study entails a modest number of chronic conditions, including the core chronic conditions recommended for inclusion in measures of multimorbidity [35]. Also, important chronic conditions such as obesity, anxiety and depression were included in this study.

Our study also has some limitations. The cross-sectional nature of the data prevents the examination of the temporality of the associations between socio-demographic factors and multimorbidity. The study included a limited number of morbidities, which are based on self-reports. Self-reported chronic disease status is subject to self-declaration bias due to under-reporting of diagnosis or forgetfulness [36,37]. Surveyed patients with only one or none of the listed morbidities, who were counted as having no multimorbidity in this study, may have other unlisted chronic conditions. In the interpretation of these findings, it is therefore important to note that the reported prevalence of multimorbidity is only based on the set of chronic conditions in the HQCA survey. Moreover, some individuals who report having multimorbidity may essentially be reporting a single chronic condition and its symptom, e.g. arthritis and chronic pain. This, potentially, may lead to over-estimation of the true prevalence of multimorbidity. It is also possible that some important groups, such as immigrants (e.g. due to language barriers), were under-sampled. A further limitation of this study is the absence of an indicator of disease severity, as provided in the Kaplan Index, the Index of Coexisting Diseases, Charlson Index or the Cumulative Illness Rating Scale [38]. Some studies have characterized conditions such as hypertension, high cholesterol and obesity as risk factors, rather than as chronic conditions [11]. A further step may be to incorporate such differences in the analysis, while weighting conditions by severity.

## Conclusions

Given the increasing prevalence of multimorbidity, understanding the prevalence and patterns of multimorbidity is important to help guide clinical care [39]. In this research, we have described the epidemiology of multimorbidity and characterized common patterns of multimorbidity in a cross-sectional sample of the adult Alberta population. We found that multimorbidity was not limited to the elderly, and was associated with sex, age, family structure and household income. The information may be vital in designing guidelines and strategies for prevention and care for people with multimorbidity. Further studies are needed to examine the consequences of multimorbidity, and to evaluate interventions to improve care of people with multimorbidity.

## Competing interests

The authors declare that they have no competing interests.

## Authors' contributions

ACB conception and design, statistical analysis and interpretation of data, drafting manuscript, revision of manuscript. DL conception and design, interpretation of data, critical revision of manuscript. ML: data acquisition, survey instrument and design, critical revision of manuscript. TC data acquisition, survey instrument and design, critical revision of manuscript. JAJ conception and design, data acquisition and interpretation of data, critical revision of manuscript. All authors read and approved the final manuscript.

## Pre-publication history

The pre-publication history for this paper can be accessed here:

http://www.biomedcentral.com/1471-2458/12/201/prepub
